# Response to “Repeating the errors of the past: the hazards of a commercial human milk industry” Modi (2024) from the Global Alliance of Milk Banks and Associations

**DOI:** 10.1038/s41390-024-03259-4

**Published:** 2024-05-28

**Authors:** Anna Coutsoudis, Rudolf Ascherl, Enrico Bertino, Nadia Garcia-Lara, Guido Moro, Sushma Nangia, Jean-Charles Picaud, Natalie Shenker, Marta Staff, Aleksandra Wesolowska, Gillian Weaver

**Affiliations:** 1 HMBASA (Human Milk Banking Association of South Africa); School of Clinical Medicine, University of KwaZulu-Natal, Durban, South Africa; 2 Frauenmilchbank-Initiative e.V., Hamburg, Germany; 3 University of Turin (ret), Italian Association of Human Milk Banks (AIBLUD); Biomedia, Via Libero Temolo No 4, 20126 Milan, Italy; 4 Department of Neonatology, 12 Octubre Hospital Regional Milk Bank, Madrid, Spain; 5 Italian Association of Donated Human Milk Banks (AIBLUD); Biomedia, Via Libero Temolo No 4, 20126 Milan, Italy; 6 National Human Milk Bank, Department of Neonatology, Lady Hardinge Medical College & Kalawati Saran Children’s Hospital, New Delhi, 110001 India; 7 Department of Neonatology, Hôpital de la Croix-Rousse, Hospices civils de Lyon, and Head of French Human Milk Bank Association, Lyon, France; 8 Department of Surgery and Cancer, Imperial College London, London, W12 0HS UK; 9 Human Milk Foundation, Gossoms End NHS Health Centre, Victory Road, Berkhamsted, HP4 1DL UK; 10 The Centre for Simulation, Analytics and Modelling (CSAM), University of Exeter Business School, Exeter, EX4 4PU UK; 11 Laboratory of Human Milk and Lactation Research, Department of Medical Biology, Medical University of Warsaw, Warsaw, Poland

Mother’s own milk is the first choice in preterm infant feeding. When mother’s milk is not available or is insufficient, donor human milk (DHM) from a well-established Human Milk Bank (HMB) is recommended by scientific organizations including the World Health Organisation (WHO), the American Academy of Pediatrics (https://www.aap.org/), and the European Society of Pediatric Gastroenterology Hepatology and Nutrition (http://www.espghan.org/).

As the Global Alliance of Human Milk Banks and Associations (GAMBA), a global collaborative network, we would like to concur with the following points made by Professor Modi in her commentary of the harms of human milk commercialization:^1^

1. It is essential to advocate against non-evidence based marketing of human milk products by for-profit companies. Unsubstantiated health claims can influence doctors and families to purchase their products, thereby potentially undermining breastfeeding of infants by their own mothers.^2^
2. We need to continue to be vigilant that DHM is not perceived as equivalent to mother’s own milk. Except in the rare cases where not possible, DHM should certainly be conceived only as a bridge until the mother has sufficient milk, given the superiority of mother’s own milk.^3^

We would however seek to differentiate non-profit human milk banking from commercialized companies. Differences abound between sectors in numerous ways, but most critically in the support of the milk donors and the underpinning commitment of milk banks to the protection and support of maternal lactation and breastfeeding. Milk banks have an ethical obligation to safeguard the health of women who donate their milk, and to ensure that DHM is predominantly used as a bridge to breastfeeding. Commercialization of human milk products introduces financial drivers that undermine both ethical obligations.

Furthermore, human milk (HM) fortifiers are currently solely produced by for-profit companies using large volumes of milk. Despite a number of trials showing no impact of HM fortifiers on supporting growth or reducing NEC impact, feed intolerance, late-onset sepsis or death,^4,5^ their ongoing production reduces the pool of milk donors available to non-profit milk banks, leading to the ongoing unequitable supply of DHM for vulnerable infants and absorbing valuable financial resource that could be invested by NICUs into lactation support.

We believe that some of Modi’s points need clarification:

1. Modi asserts that “safe formulae are now available”. We cannot agree with this broad sweeping statement as formula feeding is associated with risk compared to the gold standard of exclusive breastfeeding with mother’s own milk, particularly in resource-limited settings. Formula feeding is suboptimal in terms of the risk of necrotizing enterocolitis (NEC), poorer cognitive development, and generally increased morbidity and mortality. Infants that receive formula feeds are more likely to gain weight rapidly, but this may not be the most important marker of positive health outcomes in this population, with a growing emphasis instead on body length and head circumference as markers for appropriate growth.
2. There is insufficient evidence to support Modi’s suggestions about the possibility of harm from supplementing mother’s own milk with DHM compared to supplementing with formula milk. Her own analysis study from UK population data^6^, which she relies on heavily for this assumption, has been criticized by other authors for concerns about the integrity of the data and the methods of analysis.^7^ Although the jury is still out on the neurodevelopmental outcomes between infants fed DHM versus formula feeds there is considerable evidence that feeding preterm infants with DHM is associated with a decreased risk of NEC, most evident when data are meta-analyzed or systematically reviewed.^8–12^ Additionally, a recently published RCT of a study conducted in the US (the MILK trial) has confirmed this evidence.^13^
3. The belief by Modi that “mothers are faced with having to express milk for a prolonged period which is difficult and stressful” is a context-specific statement. Stress is reduced in hospitals with high quality lactation support that ensures mothers can express and/or feed frequently alongside hospital facilities that enable mothers to stay with their infant and provide kangaroo care.^14^ Parents need to be fully informed of the risk/benefit ratio of maternal milk versus both formula and donor milk, and that outcomes will be better if infants are fed with maternal milk.

In terms of effect on maternal lactation, supplementation with DHM, accompanied by a strong lactation support, has been associated with later increased volumes of expressed MOM and an increase in any breastfeeding at discharge.^15–18^ These data showing a positive impact on DHM use on breastfeeding rates suggest that human milk banking and the use of DHM in NICUs support the culture of breastfeeding and may serve also as a tool for promotion of lactation.

Furthermore, despite heat-induced alterations to the bioactive components of breastmilk, pasteurized milk maintains a significant degree of bacteriostatic, maturative and immune-stimulating properties.^19^ The preserved biological activity contributes to the improved outcomes observed in preterm infants fed DHM compared to infant formula. The biological plausibility of human milk (either fresh or pasteurized) justifies its use over infant formulas.

In conclusion, Modi has correctly signaled an alarm about the hazards of a commercial human milk industry. While we agree with this, we need to go further and question how the international community minimizes the hazards of a commercial human milk industry? Acknowledging the tendency of corporate organizations to develop and market products, often accompanied by aggressive and potentially misleading marketing strategies to stimulate demand, it is crucial to draw parallels with the protections created by the WHO International Code of Marketing of Breastmilk Substitutes. Policymakers globally need to understand how regulations can be further strengthened and enhanced to counter the new reality of commercialized human milk products, separate from the activity of the non-profit milk banking sector. Considering the growing number of commercial entities entering the market, potentially exploiting vulnerable individuals (both donors, infants and recipients), there is an urgent need for regulatory and ethical oversight to safeguard public interest and health.

We believe this potential for exploitation can be minimized by ensuring that every hospital invests in high quality, experienced, compassionate, respectful support for exclusive breastfeeding, especially in the NICU, before or alongside attempts to introduce DHM. The goal should always be to support the mother to produce enough of her own milk so that she is able to take over supplying milk for her infant and leaves the NICU exclusively breastfeeding.
