# Correction: Response to “Repeating the errors of the past: the hazards of a commercial human milk industry” Modi (2024) from the Global Alliance of Milk Banks and Associations

**DOI:** 10.1038/s41390-024-03415-w

**Published:** 2024-07-29

**Authors:** Anna Coutsoudis, Rudolf Ascherl, Enrico Bertino, Nadia Garcia-Lara, Guido Moro, Sushma Nangia, Jean-Charles Picaud, Natalie Shenker, Marta Staff, Aleksandra Wesolowska, Gillian Weaver

**Affiliations:** 1 HMBASA (Human Milk Banking Association of South Africa); School of Clinical Medicine, University of KwaZulu-Natal, Durban, South Africa; 2 Frauenmilchbank-Initiative e.V., Hamburg, Germany; 3 University of Turin (ret), Italian Association of Human Milk Banks (AIBLUD); Biomedia, Milan, Italy; 4 Department of Neonatology, 12 Octubre Hospital Regional Milk Bank, Madrid, Spain; 5 Italian Association of Donated Human Milk Banks (AIBLUD); Biomedia, Milan, Italy; 6 National Human Milk Bank, Department of Neonatology, Lady Hardinge Medical College & Kalawati Saran Children’s Hospital, New Delhi, India; 7 Department of Neonatology, Hôpital de la Croix-Rousse, Hospices civils de Lyon, and Head of French Human Milk Bank Association, Lyon, France; 8 Department of Surgery and Cancer, Imperial College London, London, UK; 9 Human Milk Foundation, Gossoms End NHS Health Centre, Berkhamsted, UK; 10 The Centre for Simulation, Analytics and Modelling (CSAM), University of Exeter Business School, Exeter, UK; 11 Laboratory of Human Milk and Lactation Research, Department of Medical Biology, Medical University of Warsaw, Warsaw, Poland

Correction to: *Pediatric Research* 10.1038/s41390-024-03259-4, published online 28 May 2024

The author name Rudolf Ascherl was incorrectly given as ‘Rusi Ascherl’ in the original version. Furthermore, the affiliation of the author Aleksandra Wesolowska has been corrected from “Human Milk Bank Foundation, Holy Hospital, Medical University of Warsaw, Warsaw, Poland” to Laboratory of Human Milk and Lactation Research, Department of Medical Biology, Medical University of Warsaw, Warsaw, Poland”. The original article has been corrected.

